# Risk factors for rebleeding and long-term outcomes in patients with head and neck cancer bleeding: a multicenter study

**DOI:** 10.1186/s12885-022-09945-y

**Published:** 2022-08-02

**Authors:** Chih-Kai Wang, Che-Fang Ho, Kuang-Yu Niu, Chia-Chien Wu, Yun-Chen Chang, Chien-Han Hsiao, Chieh-Ching Yen

**Affiliations:** 1 Department of Emergency Medicine, Linkou Branch, Chang Gung Memorial Hospital, Taoyuan, Taiwan; 2 Department of Otolaryngology Head and Neck Surgery, Chang Gung Memorial Hospital, Keelung, Taiwan; 3 Department of Emergency Medicine, Keelung Branch, Chang Gung Memorial Hospital, Keelung, Taiwan; 4 Department of Medical Imaging and Intervention, Linkou Branch, Chang Gung Memorial Hospital, Taoyuan, Taiwan; 5 Department of Otolaryngology Head and Neck Surgery, Linkou Branch, Chang Gung Memorial Hospital, Taoyuan, Taiwan; 6 Department of Linguistics, Indiana University, Bloomington, IN USA; 7 Institute of Emergency and Critical Care Medicine, National Yang Ming Chiao Tung University, Taipei, Taiwan

**Keywords:** Rebleeding, Head and neck cancer, Risk factor, Outcome

## Abstract

**Background:**

Acute, catastrophic bleeding in patients with head and neck cancer (HNC) is challenging and also a burden for their families and frontline physicians. This study analyzed the risk factors for rebleeding and long-term outcomes in these patients with HNC.

**Methods:**

Patients who presented to the emergency department (ED) with HNC bleeding were enrolled in this study (*N *= 231). Variables of patients with or without rebleeding were compared, and associated factors were investigated using Cox’s proportional hazard model.

**Results:**

Of the 231 patients enrolled, 112 (48.5%) experienced a recurrent bleeding event. The cumulative rebleeding incidence rate was 23% at 30 days, 49% at 180 days, and 56% at 1 year. Multivariate Cox regression analyses demonstrated that overweight-to-obesity (HR = 0.52, 95% CI 0.28–0.98, *p* = 0.043), laryngeal cancer (hazard ratio [HR] = 2.13, 95% confidence interval [CI] 1.07–4.23, *p* = 0.031), chemoradiation (HR = 1.49, 95% CI 1.001–2.94, *p* = 0.049), and second primary cancer (HR = 1.75, 95% CI 1.13–2.70, *p* = 0.012) are significant independent predictors of rebleeding, and the prognostic factors for overall survival included underweight (HR = 1.89, 95% CI 1.22–2.93, *p* = 0.004), heart rate > 110 beats/min (HR = 1.58, 95% CI 1.04–2.39, *p* = 0.032), chemoradiation (HR = 2.31, 95% CI 1.18–4.52, *p* = 0.015), and local recurrence (HR = 1.74, 95% CI 1.14–2.67, *p* = 0.011).

**Conclusions:**

Overweight-to-obesity is a protective factor, while laryngeal cancer, chemoradiation and a second primary cancer are risk factors for rebleeding in patients with HNC. Our results may assist physicians in risk stratification of patients with HNC bleeding.

## Introduction

Head and neck cancer (HNC) refers to a heterogeneous disease that includes cancers involving the oral cavity, pharynx, and larynx. On average, patients with HNC have a 5-year survival rate of ~ 60% [1]. However, they usually emerge at a late stage, with a 5-year survival rate of less than 25% for stage IV squamous cell carcinoma despite aggressive treatment [2]. Traditionally, alcohol drinking and tobacco smoking are the most important risk factors for HNC. Different from Western countries, the unique habit of betel nut chewing with or without tobacco is popular in Taiwan, which is also a well-established risk factor for HNC [3]. These patients tend to have tumor recurrence and poor prognosis compared with those who do not chew betel nut [3]. Betel nut chewing has been implicated in precipitating increased risk of HNC bleeding [4].

Acute bleeding from the head and neck area is one of the most life-threatening complications that may occur in patients with HNC. Several predisposing factors have been reported to contribute to HNC bleeding, the main factors being surgery (e.g., radical neck dissection), chemoradiation, wound infection, poor nutritional status, presence of a pharyngocutaneous fistula, tumor recurrence, and a higher T stage [5]. Historically, deconstructive therapy in the form of surgical ligation has been the most widely used treatment. Emergent surgical intervention can result in a neurological complication rate of 60% and an overall mortality rate of 40% [2]. In recent years, radiological intervention, such as deconstruction using endovascular embolization or reconstruction with covered stents, has become the firstline treatment for HNC bleeding if angiography shows contrast media extravasation, pseudoaneurysm, and irregular artery contours [1, 2, 6].

Despite improved treatment modalities, the outcomes in patients with HNC bleeding remain dismal due to acute clinical severity and advanced cancer stage [4, 7, 8]. HNC bleeding is a significant source of psychological burden for patients and their families, and acute, catastrophic bleeding can be challenging for frontline physicians [9, 10]. Prior studies have evaluated clinical outcomes in patients with HNC bleeding [4, 7, 8, 11–15]. However, until now, no studies have analyzed risk factors for patients who are at increased risk of rebleeding. This study aimed to examine the risk factors for rebleeding and the predictors of long-term overall survival in patients with index HNC bleeding.

## Materials and methods

### Study design and setting

This was a retrospective, multicenter, observational study using regularly collected electronic medical records (EMRs). The study site was the emergency department (ED) of five hospitals using the same EMR system in Taiwan, including two tertiary medical centers and three regional hospitals. The study site’s total capacity was more than 9000 beds and an annual ED visit by 500,000 patients. The study was performed in accordance with the Declaration of Helsinki and with the approval of the Chang Gung Medical Foundation institutional review board (IRB no. 202102021B0).

All adult patients with HNC who met the inclusion criteria from January 1, 2015, to December 31, 2016, were eligible for enrollment. Patients were followed-up regularly until the date of death or the date on which they were last evaluated. The last follow-up date of all patients was September 30, 2021.

### Patient selection and data collection

First, we used EMRs to identify all adult patients with the *International Classification of Diseases Tenth Revision* (ICD-10) codes of HNC (C00-C14 and C30-C32) who were treated in the ED during the study period. Second, we determined eligible patients using the following keywords: bleeding, hemorrhage, and carotid blowout. Patients with inadequate EMRs, duplicate ED visit data, and bleeding events originating from other sites, such as gastrointestinal bleeding, intracranial hemorrhage, and vaginal bleeding, were excluded. Two patients with nasal bleeding were excluded after being examined by otolaryngologists as the bleeding was deemed unrelated to HNC. The patients selected by EMRs were reviewed by two physicians (authors Chih-Kai Wang and Chieh-Ching Yen).

Clinical data, such as age, sex, body mass index (BMI), hypertension, diabetes mellitus, lifestyle factors, vital signs, white blood cell count, hemoglobin, platelet count, international normalized ratio, creatinine, alanine aminotransferase, and imaging modalities, were collected at the time of the first HNC bleeding in the ED. Information about HNC included the primary cancer site, the tumor–node–metastasis (TNM) stage according to the American Joint Committee on Cancer 7th edition, the pathologic type, cancer treatment modality, local recurrence, and a second primary cancer.

If treatment with local compression and packing failed in patients with active, life-threatening HNC bleeding, computed tomography (CT) angiography was performed. The underlying bleeding mechanism was tumor-related cause, pseudoaneurysm, postoperative complication, or fistula formation. Tumor-related causes included contrast extravasation, hypervascular tumor staining, or great vessel involvement on CT angiography or bleeding from a necrotic wound of the tumor confirmed by otolaryngologists with or without fiberoptic endoscopy. Pseudoaneurysm and fistula formation were confirmed by CT angiography.

Treatment methods for patients with HNC bleeding, that is, supportive care, endovascular therapy, or surgical intervention, were determined by a multidisciplinary team comprising otolaryngologists, oncologists, interventional radiologists, and emergency physicians. Supportive care consisted of medication with oral or intravenous tranexamic acid, epinephrine-soaked gauze packing, or observation. Endovascular therapy consisted of transarterial embolization and covered stent graft placement for bleeding involving the common carotid artery, internal carotid artery, or external carotid artery, including its branches. Surgical intervention consisted of surgical ligation and primary repair. Data of patients who required blood transfusion were also recorded. The primary outcome was a rebleeding event, and the secondary outcome was overall survival. Rebleeding was defined as the recurrence of bleeding after index HNC bleeding recorded in each EMR.

### Statistical analysis

Clinical data, presentations of cancer, bleeding characteristics, and treatment modality are shown as numbers (percentages) for categorical variables and the mean ± standard deviation (SD) for continuous variables. Comparisons between patients with or without a rebleeding event were made using a chi-square test or Fisher’s exact test, where appropriate, for categorical variables and independent Student’s *t*-tests or Mann–Whitney *U*-tests for continuous variables, depending on the distribution characteristics of the variables.

To determine independent risk factors for rebleeding and overall survival, all relevant variables that showed a significant association on univariate analysis were further analyzed using the multivariate Cox proportional hazards model. Cumulative survival curves were generated using Kaplan–Meier curve analysis and compared using the log-rank test.

All statistical analyses were performed using SPSS Statistics version 26 (SPSS Inc., Chicago, IL, USA), and a two-sided *p*-value of < 0.05 was considered statistically significant.

## Results

### Patient characteristics

**Fig. 1:**
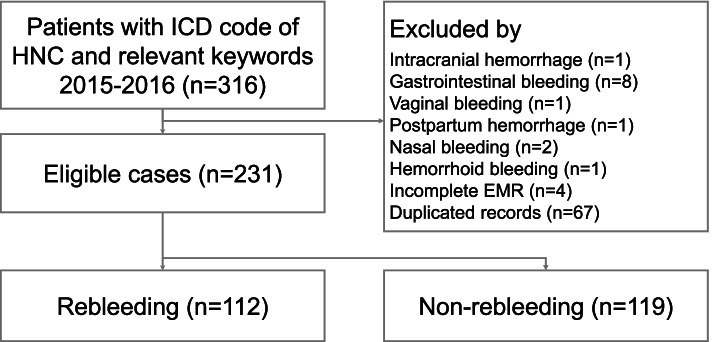
Flow chart of patient selection

A total of 231 patients with HNC met the inclusion criteria and were included in the study (Fig. 1). Table 1 summarizes the clinical characteristics of patients stratified by the rebleeding status. The mean age was 56.7 ± 10.9 years, and the BMI was 21.5 ± 3.95 kg/m^2^, with 215 (93.1%) patients being men. Of the 231 patients, 135 (58.4%) were hospitalized, 90 (39%) were discharged from the ED, and 6 (2.6%) died in the ED. In addition, 30 (13%) patients died during hospitalization. The most common cancer sites for patients with HNC bleeding were the oral cavity (*n* = 94, 40.7%), followed by the nasopharynx (*n* = 45, 19.5%), hypopharynx (*n* = 42, 18.2%), oropharynx (*n* = 34, 14.7%), and larynx (*n* = 16, 6.9%). More than half the patients were at an advanced HNC stage, and almost all patients had pathologically confirmed squamous cell carcinoma (*n* = 230, 99.6%). In addition, 92 (39.8%) patients had local recurrence, while 63 (27.3%) had a second primary cancer.

**Table 1:** Characteristics of patients with head and neck cancer bleeding according to rebleeding status

Variable	Total *N* = 231	Rebleeding *N* = 112	Non-rebleeding *N* = 119	*P* value
Age (year)	56.7 $\pm$ 10.9	55.4 $\pm$ 10.0	58.0 $\pm$ 11.5	0.073
Male	215(93.1)	106(94.6)	109(91.6)	0.362
BMI	21.5 $\pm$ 3.9	21.4 $\pm$ 3.9	21.6 $\pm$ 4.0	0.743
Systolic blood pressure (mmHg)	139.7 $\pm$ 35.5	140.3 $\pm$ 34.0	139.2 $\pm$ 37.0	0.807
Diastolic blood pressure (mmHg)	84.9 $\pm$ 18.2	85.5 $\pm$ 18.3	84.4 $\pm$ 18.2	0.643
Heart rate (beats/min)	102.6 $\pm$ 22.5	102.4 $\pm$ 22.2	102.8 $\pm$ 22.9	0.893
Smoking history	185(80.1)	96(85.7)	89(74.8)	0.038^*^
Betel nut chewer	157(68.0)	77(68.8)	80(67.2)	0.804
Hypertension	84(36.4)	38(33.9)	46(38.7)	0.455
Diabetes mellitus	51(22.1)	20(17.9)	31(26.1)	0.133
**Laboratory exam**				
WBC (10^3^/$\mathrm{\mu l}$)	11.5 $\pm$ 8.2	12.1 $\pm$ 10.1	11.0 $\pm$ 6.0	0.297
Hb (g/dl)	10.8 $\pm$ 2.3	10.7 $\pm$ 2.2	10.8 $\pm$ 2.3	0.659
PLT (10^3^/$\mathrm{\mu l}$)	264 $\pm$ 113	268 $\pm$ 116	262 $\pm$ 111	0.708
INR	1.13 $\pm$ 0.15	1.12 $\pm$ 0.10	1.14 $\pm$ 0.18	0.183
Creatinine (mg/dL)	1.08 $\pm$ 1.15	0.99 $\pm$ 1.12	1.16 $\pm$ 1.17	0.251
ALT (U/L)	41.9 $\pm$ 114	32.2 $\pm$ 45.1	51.3 $\pm$ 154	0.258

Count data are expressed as number (percentage) and continuous values are expressed as mean ± SD

*BMI* Body mass index, *WBC* White blood cell, *Hb* Hemoglobin, *PLT* Platelet count, *INR* International normalized ratio, *ALT* Alanine aminotransferase﻿

﻿*P value < 0.05

With regard to initial cancer treatment, 117 of 231 (50.6%) patients underwent surgical resection; of them, 100 (43.3%) underwent concomitant neck dissection and 80 (34.6%) concurrent flap reconstruction. Additionally, 179 of 231 (77.5%) patients underwent primary or adjuvant concurrent chemoradiotherapy, whereas 20 (8.7%) patients had no cancer-associated treatment, given either perceived concerns regarding treatment side effects or not-yet-arranged therapy at the time of diagnosis.

Comparison between patients with or without rebleeding showed that compared with the nonrebleeding group, the rebleeding group had a significantly higher proportion of smoking history (85.7% vs. 74.8%; *p* = 0.038) and a nonsignificantly higher proportion of second primary cancer (33% vs. 21.8%; *p* = 0.056). When patients were stratified by second primary cancer, patients with second primary cancer had a significantly more frequent rate of chemoradiation compared with those without a second primary cancer (87.3% vs. 73.8%; *p* = 0.029) (Table 2).

**Table 2:** Features of cancer and rebleeding in patients with head and neck cancer according to rebleeding status

Variable	Total *N* = 231	Rebleeding *N* = 112	Non-rebleeding *N* = 119	*P* value
**Cancer site**				0.235
Oral cavity	94(40.7)	41(36.6)	53(44.5)	
Nasopharynx	34(14.7)	16(14.3)	18(15.1)	
Oropharynx	42(18.2)	20(17.9)	22(18.5)	
Hypopharynx	45(19.5)	23(20.5)	22(18.5)	
Larynx	16(6.9)	12(10.7)	4(3.4)	
**Initial T stage**				0.371
T1	27(11.7)	10(8.9)	17(14.3)	
T2	36(15.6)	21(18.8)	15(12.6)	
T3	27(11.7)	15(13.4)	12(10.1)	
T4	131(56.7)	63(56.3)	68(57.1)	
Unknown	10(4.3)	3(2.7)	7(5.9)	
**Initial N stage**				0.590
N0	108(46.8)	52(46.4)	56(47.1)	
N +	112(48.5)	58(51.8)	54(45.4)	
Unknown	11(4.8)	2(1.8)	9(7.6)	
**Initial M stage**				0.116
M0	199(86.1)	102(91.1)	97(81.5)	
M1	20(8.7)	7(6.3)	13(10.9)	
Unknown	12(5.2)	3(2.7)	9(7.6)	
**Pathology type**				0.540
Squamous cell carcinoma				
Keratinizing carcinoma	213(92.2)	104(92.9)	109(91.6)	
Nonkeratinizing carcinoma	16(6.9)	7(6.3)	9(7.6)	
Sarcomatoid carcinoma	1(0.4)	0(0)	1(0.8)	
Adenocarcinoma	1(0.4)	1(0.9)	0(0)	
**Cancer treatment**				
Surgical resection	117(50.6)	57(51.4)	60(50.4)	0.888
Chemoradiation	179(77.5)	92(82.1)	87(73.1)	0.100
Neck dissection	100(43.3)	50(45.0)	50(42.7)	0.725
Flap reconstruction	80(34.6)	42(37.8)	38(32.5)	0.397
**Local recurrence**	92(39.8)	50(45.0)	42(36.2)	0.175
**Second primary cancer**	63(27.3)	37(33.0)	26(21.8)	0.056
**Bleeding cause**				0.726
Tumor-related	183(79.2)	91(81.3)	92(78.6)	
Pseudoaneurysm	25(10.8)	12(10.7)	13(10.9)	
Postoperative complication	22(9.5)	9(8)	13(10.9)	
Fistula formation	1(0.4)	0(0)	1(0.8)	
**Bleeding type**				0.509
Self-limited	100(43.3)	46(41.1)	54(45.4)	
Active bleeding	131(56.7)	66(58.9)	65(54.6)	
**Emergent CTA**	76(32.9)	41(36.6)	35(29.4)	0.245
**Bleeding treatment**				0.523
Supportive care	191(82.7)	92(82.1)	99(83.2)	
Embolization	31(13.4)	16(14.3)	15(12.6)	
Covered stent	5(2.2)	1(0.9)	4(3.4)	
Surgical ligation	3(1.3)	2(1.8)	1(0.8)	
Primary repair	1(0.4)	1(0.9)	0(0)	
**Blood transfusion**	98(42.4)	49(43.8)	49(41.2)	0.692

Count data are expressed as number (percentage) and continuous values are expressed as mean ± SD

*CTA* Computed tomography angiography

### Bleeding and various treatment modalities

In the ED, 131 of 231 (56.7%) patients had active bleeding, while the remaining patients (*n* = 100, 43.3%) had self-limited bleeding. The underlying mechanisms of HNC bleeding were as follows: tumor-related (*n* = 183, 79.2%), pseudoaneurysm (*n* = 25, 10.8%), postoperative complications (*n* = 22, 9.5%), and fistula formation (*n* = 1, 0.4%). Less than half of the patients (*n* = 76, 32.9%) underwent emergent CT angiography to confirm the bleeder location. Of these, 7 (9.2%) patients showed contrast extravasation on imaging (7 from the external carotid artery), 19 (25%) had pseudoaneurysm (12 from the external carotid artery, 5 from the internal carotid artery, and 2 from the common carotid artery), 6 (7.9%) had pseudoaneurysm combined with contrast extravasation (5 from the external carotid artery and 1 from the internal carotid artery), and 44 (57.9%) had no identified vessel involvement.

With regard to bleeding treatment, most patients (*n* = 191, 82.7%) received only supportive care, while other treatments comprised transarterial embolization (*n* = 31, 13.4%), covered stent placement (*n* = 5, 2.2%), surgical ligation (*n* = 3, 1.3%), and primary repair (*n* = 1, 0.4%). In addition, 98 (42.4%) patients received blood transfusion (Table 2). The mortality rates after supportive care and transarterial embolization were 13.6% (26/191) and 12.9% (4/31), respectively. Patients who underwent covered stent placement, surgical ligation, and primary repair all survived to hospital discharge.

### Rebleeding assessment in patients with HNC

**Fig. 2:**
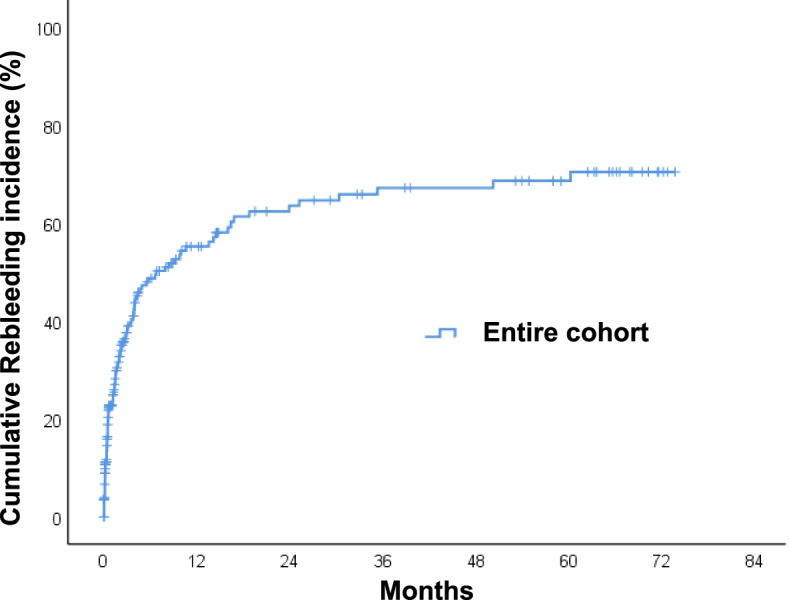
Cumulative incidence curves of rebleeding in patients with head and neck cancer

**Fig. 3:**
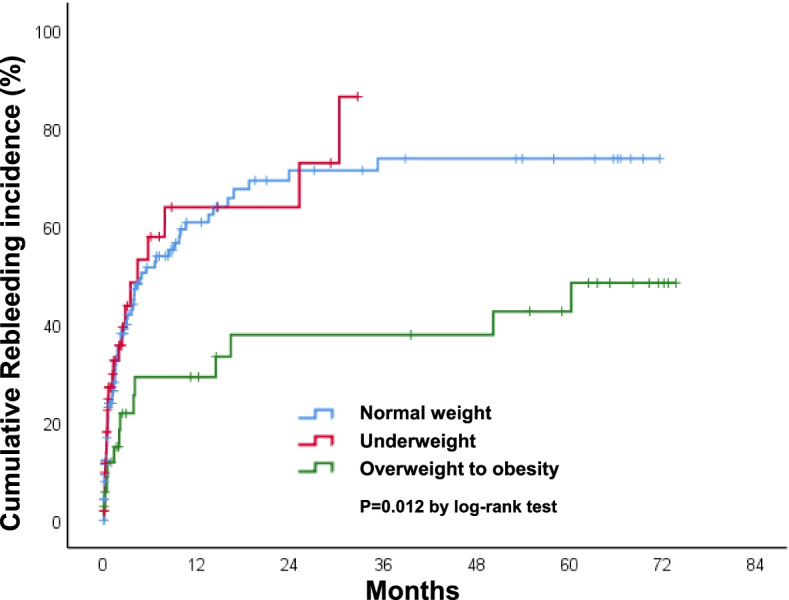
Cumulative incidence curves of rebleeding in patients with head and neck cancer stratified by BMI

Of 231 patients, 112 (48.5%) experienced a rebleeding event. The median time to rebleeding was 59 days (interquartile rage [IQR] = 13–266) for those with index HNC bleeding, and 26 (23.2%) patients died after readmission. The cumulative incidence rate of rebleeding was 23% at 30 days, 49% at 180 days, and 56% at 1 year (Fig. 2). Patients with laryngeal cancer had significantly higher rebleeding rate compared with those without (75 vs. 46.5%, *p* = 0.028). The bleeding rate stratified by cancer sites and BMI were presented in Table 3. We analyzed the influence of the BMI on survival by stratifying the patients into three groups based on the World Health Organization classification: underweight (< 18.5 kg/m^2^), normal weight (18.5 to < 25 kg/m^2^), and overweight-to-obesity (≥ 25 kg/m^2^) [16]. The overweight-to-obesity group showed the lowest cumulative rebleeding incidence curve (*p* = 0.012) (Fig. 3). Univariate and multivariate Cox regression analyses were performed to assess factors associated with increased risk of rebleeding. Univariate predictors included overweight-to-obesity (hazard ratio [HR] = 0.44, 95% confidence interval [CI] 0.24–0.80, *p* = 0.007), laryngeal cancer (hazard ratio [HR] = 2.20, 95% confidence interval [CI] 1.15–4.22, *p* = 0.017), chemoradiation (HR = 2.04, 95% CI 1.24–3.35, *p* = 0.005), and second primary cancer (HR = 1.57, 95% CI 1.06–2.33, *p* = 0.026). After adjustment, multivariate analysis showed that overweight-to-obesity (HR = 0.52, 95% CI 0.28–0.98, *p* = 0.043), laryngeal cancer (hazard ratio [HR] = 2.13, 95% confidence interval [CI] 1.07–4.23, *p* = 0.031), chemoradiation (HR = 1.49, 95% CI 1.001–2.94, *p* = 0.049), and second primary cancer (HR = 1.75, 95% CI 1.13–2.70, *p* = 0.012) were all statistically significant independent predictors of rebleeding risk (Table 4).

**Table 3:** The rebleeding rate in patients with head and neck cancer according to cancer sites and BMI

Variable	Total *N* = 231	Rebleeding *N* = 112	Rebleeding rate	*P* value
**Cancer site**				
Oral cavity	94(40.7)	41(36.6)	43.6%	0.220
Nasopharynx	34(14.7)	16(14.3)	47.1%	0.857
Oropharynx	42(18.2)	20(17.9)	47.6%	0.901
Hypopharynx	45(19.5)	23(20.5)	51.1%	0.694
Larynx	16(6.9)	12(10.7)	75.0%	0.028^*^
**BMI**				
Underweight	53(22.9)	24(21.4)	45.3%	0.595
Normal weight	143(61.9)	75(67)	52.4%	0.124
Overweight-to-obesity	35(15.2)	13(11.6)	37.1%	0.145

Count data are expressed as number (percentage) and continuous values are expressed as mean ± SD

*BMI* Body mass index

*P value < 0.05

**Table 4:** Univariate and multivariate analyses of predictors for rebleeding with Cox proportional hazards model

	Univariate		Multivariate	
	HR(95%CI)	*P* value	HR(95%CI)	*P* value
Age	0.98(0.97,1.002)	0.084		
Male	1.49(0.65,3.39)	0.343		
BMI			0.95(0.90,0.99)	0.041^*^
Underweight	1.13(0.71,1.80)	0.605	1.15(0.72,1.84)	0.570
Normal weight	Reference		Reference	
Overweight-to-obesity	0.44(0.24,0.80)	0.007	0.52(0.28,0.98)	0.043^*^
Cancer site				
Oral cavity	Reference		Reference	
Nasopharynx	1.21(0.68,2.16)	0.516	1.31(0.70,2.45)	0.400
Oropharynx	1.17(0.69,2.00)	0.563	1.37(0.80,2.35)	0.257
Hypopharynx	1.27(0.76,2.12)	0.364	1.30(0.77,2.21)	0.325
Larynx	2.20(1.15,4.22)	0.017	2.13(1.07,4.23)	0.031^*^
Smoking history	1.34(0.79,2.27)	0.282		
SBP < 90 (mmHg)	0.97(0.427,2.22)	0.950		
Heart rate > 110 (beats/min)	1.39(0.93,2.07)	0.109		
Hypertension	0.79(0.54,1.17)	0.793		
Diabetes mellitus	0.62(0.38,1.001)	0.050		
Surgical resection	0.82(0.56,1.19)	0.290		
Chemoradiation	2.04(1.24,3.35)	0.005	1.49(1.001,2.94)	0.049^*^
Neck dissection	0.92(0.63,1.33)	0.646		
Flap reconstruction	1.04(0.71,1.53)	0.830		
T stage	1.15(0.97,1.37)	0.105		
N stage	1.10(0.93,1.30)	0.279		
M stage	0.88(0.41,1.89)	0.739		
Local recurrence	1.45(0.99,2.13)	0.054		
Second primary cancer	1.57(1.06,2.33)	0.026	1.75(1.13,2.70)	0.012^*^
WBC > 11.0 (10^3^/$\mathrm{\mu l}$)	1.40(0.95,2.06)	0.088		
Hb < 10.0 (g/dl)	0.88(0.60,1.30)	0.529		
PLT < 150 (10^3^/$\mathrm{\mu l}$)	1.08(0.63,1.83)	0.787		

*HR* Hazard ratio, *95% CI* 95% confidence interval

*SBP* Systolic blood pressure

^*^P value < 0.05

### Long-term mortality and survival analysis for patients with HNC bleeding

**Fig. 4:**
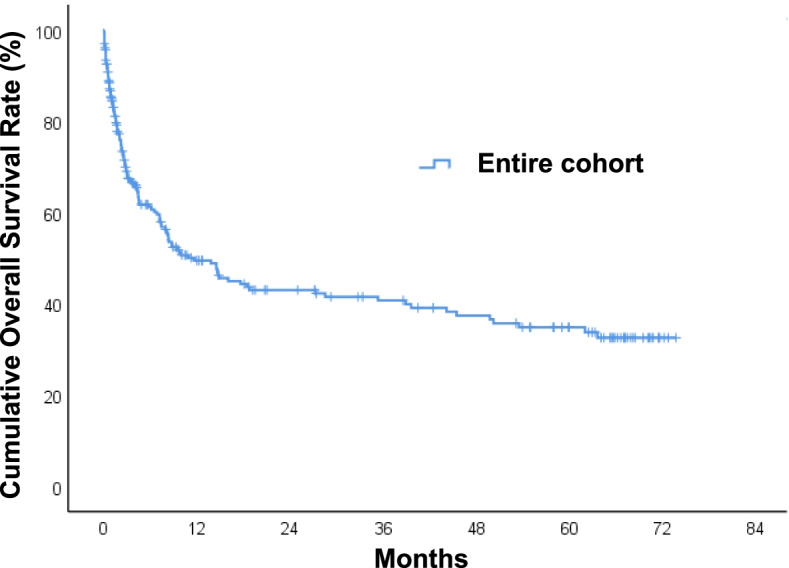
Kaplan–Meier survival curves of patients with head and neck cancer bleeding

During a median follow-up period of 5.4 months (IQR = 1.3–27.2) after a diagnosis of HNC bleeding, 125 of 231 (54.1%) patients died. The 30-day mortality rate was 15%. The overall survival at 1, 3, and 5 years was 50%, 41%, and 35%, respectively. The median survival time was 11 months (Fig. 4). Univariate and multivariate Cox regression analyses were performed to predict long-term overall survival. Univariate predictors included underweight (HR = 2.21, 95% CI 1.48–3.28, *p* < 0.001), overweight-to-obesity (HR = 0.47, 95% CI 0.26–0.85, *p* = 0.012), heart rate > 110 beats/min (HR = 2.00, 95% CI 1.38–2.88, *p* < 0.001), hypopharygneal cancer (HR = 1.83, 95% CI 1.21–2.76, *p* = 0.004), surgical resection (HR = 0.60, 95% CI 0.42–0.86, *p* = 0.005), chemoradiation (HR = 3.15, 95% CI 1.83–5.42, *p* < 0.001), T stage (HR = 1.36, 95% CI 1.14–1.63, *p* = 0.001), N stage (HR = 1.25, 95% CI 1.06–1.47, *p* = 0.008), local recurrence (HR = 1.93, 95% CI 1.35–2.76, *p* < 0.001), WBC count > 11.0 (10^3^/µL; HR = 1.48, 95% CI 1.03–2.12, *p* = 0.034), and hemoglobin < 10.0 g/dL (HR = 1.90, 95% CI 1.35–2.73, *p* < 0.001). Multivariate analyses indicated that underweight (HR = 1.89, 95% CI 1.22–2.93, *p* = 0.004), heart rate > 110 beats/min (HR = 1.58, 95% CI 1.04–2.39, *p* = 0.032), chemoradiation (HR = 2.31, 95% CI 1.18–4.52, *p* = 0.015), and local recurrence (HR = 1.74, 95% CI 1.14–2.67, *p* = 0.011) were all statistically significant independent prognostic factors of overall survival (Table 5).

**Table 5:** Univariate and multivariate analyses of predictors for long-term survival with Cox proportional hazards model

	Univariate		Multivariate	
	HR(95%CI)	*P* value	HR(95%CI)	*P* value
Age	0.99(0.97,1.003)	0.986		
Male	0.81(0.42,1.54)	0.517		
BMI				
Underweight	2.21(1.48,3.28)	< 0.001	1.89(1.22,2.93)	0.004^*^
Normal weight	Reference		Reference	
Overweight-to-obesity	0.47(0.26,0.85)	0.012	0.75(0.39,1.43)	0.379
Cancer site				
Oral cavity	Reference		Reference	
Nasopharynx	1.31(0.77,2.23)	0.312	0.95(0.47,1.90)	0.881
Oropharynx	1.26(0.76,2.11)	0.373	1.14(0.63,2.07)	0.664
Hypopharynx	2.04(1.28,3.25)	0.003	1.43(0.85,2.40)	0.179
Larynx	0.98(0.46,2.09)	0.965	0.65(0.30,1.44)	0.291
Smoking history	1.09(0.69,1.73)	0.712		
SBP < 90 (mmHg)	1.76(0.95,3.28)	0.074		
Heart rate > 110 (beats/min)	2.00(1.38,2.88)	< 0.001	1.58(1.04,2.39)	0.032^*^
Hypertension	0.72(0.49,1.04)	0.082		
Diabetes mellitus	0.77(0.50,1.19)	0.235		
Surgical resection	0.60(0.42,0.86)	0.005	0.86(0.53,1.39)	0.856
Chemoradiation	3.15(1.83,5.42)	< 0.001	2.31(1.18,4.52)	0.015^*^
Neck dissection	0.88(0.62,1.26)	0.495		
Flap reconstruction	0.96(0.66,1.39)	0.813		
T stage	1.36(1.14,1.63)	0.001	1.16(0.94,1.42)	0.162
N stage	1.25(1.06,1.47)	0.008	1.11(0.90,1.37)	0.337
M stage	1.76(0.94,3.29)	0.075		
Local recurrence	1.93(1.35,2.76)	< 0.001	1.74(1.14,2.67)	0.011^*^
Second primary cancer	1.37(0.93,2.00)	0.108		
WBC > 11.0 (10^3^/$\mathrm{\mu l}$)	1.48(1.03,2.12)	0.034	1.31(0.88,1.96)	0.190
Hb < 10.0 (g/dl)	1.90(1.35,2.73)	< 0.001	1.15(0.75,1.76)	0.530
PLT < 150 (10^3^/$\mathrm{\mu l}$)	1.22(0.76,1.96)	0.401		

*HR* Hazard ratio, *95% CI* 95% Confidence interval

^*^P value < 0.05

## Discussion

To the best of our knowledge, this is the first cohort study to identify predictors of rebleeding in patients with HNC. The major findings of the study were as follows: (1) the rebleeding rate was 48.5%, and the median time to rebleeding was 59 days; (2) the in-hospital mortality rate was 13%, and the long-term median survival time was 11 months; (3) increased BMI (HR = 0.95, *p* = 0.041) was a significant independent protective factor, while chemoradiation (HR = 1.68, *p* = 0.049) and a second primary cancer (HR = 1.60, *p* = 0.023) were risk factors for increased risk of rebleeding in patients with HNC; and (4) increased BMI (HR = 0.93, *p* = 0.011), heart rate > 110 beats/min (HR = 1.54, *p* = 0.040), chemoradiation (HR = 2.35, *p* = 0.010), and local recurrence (HR = 1.70, *p* = 0.014) were significant independent prognostic factors of overall survival.

Previous studies have invested multiple aspects of HNC bleeding, such as the bleeding rate, survival rate, and management, while the risk factors for rebleeding have not been identified. In the existing research, significant bleeding is found in 6%–14% of patients with HNC [1], and massive bleeding accounts for 6%–10% of patients with advanced HNC [4]. Acute catastrophic bleeding in the head and neck area may lead to a life-threatening situation, which involves not only hemorrhagic shock but also aspiration of blood, contributing to asphyxiation. Advanced HNC with the involvement of major vasculature implies poor prognosis, with an overall survival rate ranging from 35%–50% [12]. One of the reasons for high mortality is that management of unexpectedly massive HNC bleeding is complicated and difficult for frontline physicians. Massive bleeding necessitates immediate intervention, but it is usually hampered by hemodynamic instability, decreased visualization, and difficulty in localizing the bleeding source. Most previous studies have focused on the management of and outcome in carotid blowout syndrome (CBS) [4, 8, 17–21]. Despite the extensive research, no studies have investigated who are at increased risk of rebleeding when the first episode of HNC bleeding occurs. The reason there are no identified risk factors for HNC rebleeding is that previous studies included a relatively small number of patients (< 50) [7, 8, 11, 12, 17, 22] and could not detect an independent predictor. Additionally, CBS accounts for only a portion of HNC bleeding, and such studies may preclude a large majority of patients with HNC bleeding. Our study adds to the existing literature and will help physicians identify patients at risk in a real-world setting.

The current study identified an association between BMI and rebleeding rate as well as long-term survival rate. We found that an increased BMI is associated with a lower incidence of rebleeding events. The overweight-to-obesity group had a significantly lower cumulative event incidence curve compared with the normal weight and underweight groups. Chen et al. retrospectively reviewed patients with HNC and identified BMI < 22.5 kg/m^2^ as an independent risk factor for CBS development; however, the authors did not investigate the effect of BMI on rebleeding risk [13]. The BMI is considered a biomarker reflecting nutritional status and is widely used since it is easy to measure precisely. Malnutrition is common in patients with advanced cancer, with a prevalence rate of 30%–85% [23]. Patients with advanced HNC tend to be malnourished, with an impaired calorie and protein balance and weakening of immune defense [24]. A poor nutritional status puts patients with HNC at an increased risk of CBS and has prognostic influence on outcomes in these patients [13, 22]. One possible explanation is that malnutrition results in less soft-tissue coverage, with weakening arterial walls in the cervical region [22]. Another proposed mechanism is the interaction between nutritional status and the immune system [25]. Malnutrition reduces the resistance to infection by depressing the immune system and may further trigger a vicious cycle, exacerbating the poor nutritional status and inflammatory process. These combined effects render the carotid artery or its branches vulnerable to vasa vasorum thrombosis, poor intravascular wound healing, and arterial wall injury [22, 26]. In this study, a decreased BMI was also a predictive factor of poor long-term overall survival in patients with HNC bleeding. One large prospective cohort study by Gama et al. demonstrated that underweight is an adverse prognostic factor, while overweight has better prognosis in patients with HNC [24]. Underweight patients may have more comorbidities, an advanced cancer stage, and poorer nutritional status. They also tend to have lower tolerance to the toxicities of cancer treatment [24, 27]. As shown in this study, the adverse impact of a low BMI exerts an additive effect, with acute hemorrhage in patients with HNC, leading to a worse overall survival.

Our study demonstrated that chemoradiation and a second primary cancer were associated with an increased risk of rebleeding. Although chemotherapy and radiotherapy limit cancer progression and prolong the life expectancy of patients with HNC, they are also accompanied by an up to 7.6 times higher risk of bleeding [5]. Studies have indicated that radiotherapy results in upregulation of pro-inflammatory cytokines and inflammatory cells are recruited to the area of vascular injury [28, 29]. Radiation causes further free-radical production and oxidative stress in affected tissues, contributing to thrombosis and obliteration of the adventitial vasa vasorum, adventitial fibrosis, premature atherosclerosis, and arterial wall weakening [7]. Chemotherapy-induced vascular toxicity is frequently secondary to endothelial dysfunction, which manifests as a loss of vasorelaxant effects and reduced anti-inflammatory as well as vascular reparative functions [30]. These effects make the carotid artery more vulnerable to necrosis or rupture due to ischemia [5, 28]. In addition, patients with a second primary cancer usually require re-irradiation with or without chemotherapy [31]. They may receive cumulative radiation doses totaling over 200% of the tolerance of normal tissues [32]. Re-irradiation exposes these tissues to considerable toxicity and thereby leads to a higher risk of hemorrhage [5]. A recent longitudinal study by Jacobi et al. revealed that chemotherapy is related to an increased risk of CBS development [7]. However, our study showed that not only chemoradiation but also a second primary cancer contributes to a significantly higher risk of rebleeding in patients with HNC. Our study found that patients with laryngeal cancer were significantly more likely to have rebleeding than those with HNC at other sites. We speculate that advanced laryngeal cancer requiring multiple treatments makes blood vessels more vulnerable to side effects and results in massive hemorrhage owing to blood vessel damages in the infiltrated region.

Our identification of local recurrence and chemoradiation as two other risk factors for long-term survival is consistent with the prior studies that reported the association between these factors and worse long-term survival in patients with CBS [7, 22]. Despite being at an advanced cancer stage, patients with local recurrence may need more cancer-related treatment, such as chemoradiation or operative resection, which poses an increased risk of bleeding and affects survival outcomes. Patients with HNC can present with self-limited episodes of minor bleeding followed by subsequent massive bleeding and cardiovascular collapse within hours. It is important to note that tachycardia, rather than hypotension, is an independent significant risk factor for mortality as the value of blood pressure tends to be influenced by the vessel constriction and physiological compensation—which can mask the real hemodynamic status—in the early stage of hypovolemic shock [33]. Aggressive fluid resuscitation and inotropes are the mainstay of medical therapy to restore normo-tension and reduce tachycardia. Prolonged volume depletion places patients at great risk of high morbidity and mortality since decreased cardiac output leads to insufficient tissue oxygenation that may cause multiorgan failure [34].

### Limitations

This study had several limitations. First, the study design, as a retrospective chart review, did not allow for uniform collection of all clinical variables. For example, accurate total chemoradiation dosing data were not available in one-third of our patients, limiting our analysis of the association between doses and rebleeding risk. Second, although we used the BMI as an anthropometric proxy for nutritional status, it does not consider body fat distribution, cardiorespiratory status, or other health factors like sarcopenia does. Third, this was a single-country study, and race as well as ethnicity in Taiwan is relatively homogenous. Further prospective multicountry studies are necessary to strengthen our findings.

## Conclusion

Overweight-to-obesity is a protective factor, while laryngeal cancer, chemoradiation and a second primary cancer are risk factors for rebleeding in patients with HNC. Our results may play a pivotal role in assisting physicians in risk stratification and better decision making for patients with HNC with life-threatening bleeding.

## Data Availability

The datasets generated and analyzed during the current study are not publicly available due to restrictions but are available from the corresponding author on reasonable request.
